# Role of Eu^2+^ and Dy^3+^ Concentration in the Persistent Luminescence of Sr_2_MgSi_2_O_7_ Glass-Ceramics

**DOI:** 10.3390/ma15093068

**Published:** 2022-04-23

**Authors:** Laura Fernández-Rodríguez, Rolindes Balda, Joaquín Fernández, Alicia Durán, María Jesús Pascual

**Affiliations:** 1 Ceramics and Glass Institute (CSIC), C/Kelsen 5, Campus de Cantoblanco, 28049 Madrid, Spain; laura.fernandez@icv.csic.es (L.F.-R.); aduran@icv.csic.es (A.D.); 2 Departamento Física Aplicada, Escuela Superior de Ingeniería, Universidad del País Vasco (UPV-EHU), 48013 Bilbao, Spain; rolindes.balda@ehu.eus; 3 Centro de Física de Materiales, UPV/EHU-CSIC, 20018 San Sebastian, Spain; 4 Donostia International Physics Center DIPC, 20018 San Sebastian, Spain; xuaco@dipc.org

**Keywords:** Sr_2_MgSi_2_O_7_, phosphors, europium, glass-ceramics, persistent luminescence

## Abstract

In this study, glass-ceramics based on Sr_2_MgSi_2_O_7_ phosphor co-doped with Eu/Dy were obtained from the sintering and crystallisation of glass powders. The glasses were melted in a gas furnace to simulate an industrial process, and the dopant concentration was varied to optimise the luminescence persistence times. The doped parent glasses showed red emission under UV light excitation due to the doping of Eu^3+^ ions, while the corresponding glass-ceramics showed persistent blue emission corresponding to the presence of Eu^2+^ in the crystalline environment. The dopant concentration had a strong impact on the sintering/crystallisation kinetics affecting the final glass-ceramic microstructure. The microstructures and morphology of the crystals responsible for the blue emission were observed by scanning electron microscopy–cathodoluminescence. The composition of the crystallised phases and the distribution of rare-earth (RE) ions in the crystals and in the residual glassy phase were determined by X-ray diffraction and energy dispersive X-ray analysis. The emission and persistence of phosphorescence were studied by photoluminescence.

## 1. Introduction

Rare-earth (RE)-doped glasses and glass-ceramics are considered good matrices for luminescent or light-emitting applications. Some of their well-known applications are LEDs, sensing, solar cells, biomedicine, etc. [1,2,3,4].

A wide variety of host materials are used as luminescent compounds, but most known hosts decline when it comes to persistent luminescence. For many decades, copper-doped zinc sulphide [5] has been the most widely used persistent phosphor; however, its brightness and lifetime were rather low (<1 min) for practical purposes. Currently, the use of ZnS: Cu has decreased in favour of rare-earth-doped aluminates and silicates [6].

Since the mid-1990s, a new generation of persistent luminescent phosphors has been developed and has partially entered the commercial market [7]. Most research groups focused their attention on strontium aluminate matrices doped with europium and its derivatives SrAl_2_O_4_:Eu^2+^, Dy^3+^ [8]. Eu^2+^-, Dy^3+^-, and Nd^3+^-doped aluminates exhibit blue-centred emission bands [9,10]. These aluminates have a half-life close to 6 h [11,12]. Another group of materials that has recently been investigated is silicates. Silicates were chosen as hosts due to their special properties, such as low cost, ease of preparation, and excellent thermal and chemical stabilities. The M_2_MgSi_2_O_7_ (M = Ca, Sr, Ba) family of materials, also called alkaline earth akermanites, plays a similar role to that of MAl_2_O_4_ in the aluminate group. The best-known persistent luminescent silicate, Sr_2_MgSi_2_O_7_: Eu^2+^, Dy^3+^ was investigated for the first time by Lin et al. in 2001 [13]. The emission band is centred in the blue and has a half-life of about 10 h when the material is produced by solid-state synthesis [14].

The solid-state reaction is the most common way to prepare crystalline materials based on M_2_MgSi_2_O_7_ crystalline materials, but coprecipitation [15] and combustion [16] methods have also been successfully applied.

In the investigations of Tian et al. [17], the concentration dependence and energy transfer of Y_2_(MoO_4_)_3_: Dy^3+^ type phosphors were analysed. The mechanism of energy transfers between Dy^3+^ ions was studied by several theories, and it was concluded that the electric dipole–dipole interaction between Dy^3+^ ions is the main physical mechanism of energy transfers between Dy^3+^ ions.

Jiang et al. [18] synthesised Ca_2_MgSi_2_O_7_: Eu, Dy, Nd, using the solid-state reaction method, and modified the Dy/Eu ratio, to observe the changes in the luminescent response. The emission intensity at 518 nm and the decay rate were different for phosphors with different Dy/Eu ratios. By slightly increasing the Eu^2+^ content with a constant Dy^3+^ and Nd^3+^ content (Dy/Eu = 1/2 or 1/1), both the emission intensity and afterglow increased. However, when the value of Dy/Eu was higher than 20/7, the afterglow time and emission intensity decreased, which can be attributed to concentration quenching of Eu^2+^. Nevertheless, no persistence studies have been included in their research.

Shrivastava et al. [19] prepared Ca_2_MgSi_2_O_7_: Eu^2+^, Dy^3+^ of different Eu/Dy concentration ratios with solid-state reaction. The emission spectra were identical in shape, and the bands differed only in intensities, with the highest observed for Eu/Dy ratio = 0.5/1.5. The broad emission spectra centred at 510 nm were observed under the ultraviolet excitation of 395 nm, which correspond to Eu^2+^ emission. Since the crystal field can greatly affect the electronic states, this suggests that the crystal field does not change much with compositional variations. The intensity of the thermoluminescence signals decreased, and the position of the temperature peak shifted to the upper side with increasing delay time, indicating a reasonable retraction associated with non-first order kinetics. The decay curve showed characteristics of a simple exponential equation, with a decay constant of 4.96 min.

He et al. [20] successfully prepared Sr_2_MgSi_2_O_7_:Eu^2+^, Dy^3+^ nanofibers using the electrospinning method. The Sr_2_MgSi_2_O_7_:Eu^2+^, Dy^3+^ nanofibers were formed after calcining at 1150 °C for 5 h. The optimum concentration of Eu and Dy co-doping for this investigation was x = 0.03, y = 0.04 in Sr_2-x-y_MgSi_2_O_7_:xEu^2+^, yDy^3+^; the nanofibers had a blue emission peak at 471 nm, attributed to typical Eu^2+^ emission, which is made from the 4f^6^5d^1^-4f^7^ transition. The co-doped Dy^3+^ ion plays an important energy transfer and electron trapping role and can prolong the persistence time of the luminescent Sr_2_MgSi_2_O_7_:Eu^2+^, Dy^3+^ nanofibers. However, no data regarding decay or persistence times have been provided in this study.

Luminescent emissions in glass-ceramic materials are strongly affected by crystallinity, as well as by microstructure, number of active centres present, matrix composition, crystal field, etc.

Wondraczek et al. [21] investigated (Sr,Ca)–akermanite (Ca,Sr)_2_MgSi_2_O_7_ glass-ceramics doped with europium. The red emission of the glass presented a characteristic Eu^3+^ band, while the glass-ceramic emitted in blue with the characteristic Eu^2+^ band. Scanning electron microscopy–cathode-luminescence (SEM–CL) characterisation indicated that the crystalline phase was associated with Eu^2+^ emission, and it was claimed that Eu^2+^ is incorporated in the Sr^2+^ sites of the akermanite structure, while Eu^3+^ accumulates in the intergranular phase of the glass.

According to Hölsä’s investigations [22] on the mechanism of luminescence, the exact role of defects in excitation energy storage is still not well understood. It has been established that defects can form due to charge compensation and preparation conditions. The most significant structural modifications due to defects in the environment of the Eu^2+^ luminescent centre were found with the introduction of the strontium vacancy. Electron traps were created by the Eu^2+^ and strontium vacancy, as well as by the oxygen vacancy, while strontium, magnesium, and silicon vacancies also created shallow voids in the material. Electron traps close to the conduction band may contribute to the persistent luminescence efficiency, as they are easily bleached by thermal energy at room temperature. However, traps that are too shallow or too deep can decrease this efficiency. Later, Duan [23] proposed that, for every oxygen vacancy, there are two associated electrons, and these fully occupy the singlet state, which is created from the valence band states of the host. Furthermore, the gap between oxygen and vacancy narrows on the order of 1–2 eV relative to the Sr_2_MgSi_2_O_7_ gap. This means that the incorporation of oxygen vacancies can enhance the persistent luminescence of the material by improving the absorption of ultraviolet light. Eu^2+^ ions act as luminescent centres, while Dy^3+^ ions act mainly as traps in the host [24]. The oxygen vacancies and Dy^3+^ ions provide the deep traps; the residual conduction band electrons can be trapped after turning off the UV light source. The Dy ions in Sr_2_MgSi_2_O_7_: Eu^2+^, Dy^3+^ only act as trapping centres, since Sr_2_MgSi_2_O_7_: Dy^3+^ without Eu doping has no luminescent properties [25].

As discussed extensively in a previous paper [26], glass-ceramics based on Eu/Dy-doped Sr_2_MgSi_2_O_7_ phosphor were obtained from sintering and crystallisation of glass powders (base composition 55SiO_2_-27SrO-18MgO mol%). Increasing the dopant content resulted in higher stability of the glass against crystallisation. Electric and gas furnaces were used for glass melting. The doped parent glasses showed red emission under UV light excitation, while the corresponding glass-ceramics showed blue emission. The emission spectra of the glass-ceramics indicated features attributable to Eu^2+^ and Eu^3+^ cations. The Eu/Dy co-doped glass-ceramic provided a lower Eu^3+^ associated signal than that of the Eu-doped glass-ceramic. Cathodoluminescence measurements indicated that Eu^2+^ emission originated from Sr_2_MgSi_2_O_7_ crystals, and Eu^3+^ emission from the remaining glassy phase, suggesting that europium reduction occurs in the crystalline phase. Photoluminescence emission spectra showed a main peak at 484 nm, associated with typical T_2g_→^8^S_7/2_ transitions of Eu^2+^ under excitation at 390 nm in the glass-ceramics. The presence of Dy^3+^ increased persistence in the samples melted in the gas furnace. Dy^3+^ ions are generally attributed to the formation of deeper traps and increased persistent luminescence. The results of our latest published research [27], according to which only glass-ceramics co-doped with Dy^3+^ showed some persistence, are in agreement with this hypothesis.

Analysis of the Eu L_3_-edge XANES spectra of the most persistent sample revealed that the Eu^2+^ ratio was ~4% and that Eu^2+^ was incorporated in the crystalline phase at a concentration of ~0.16 wt%. A higher amount of Eu^2+^ was detected in the co-doped samples treated in a reducing atmosphere, but this did not lead to improvement in the persistence.

We suggested that the luminescence mechanism involves the presence of shallow electron traps that are suddenly emptied for temperatures above 100 K. The most persistent luminescence is likely due to an effective concentration of Eu^2+^ in the dendritic-shaped crystals, which are formed for this particular composition, and with a suitable level of Sr vacancies, even though Sr vacancies are most likely formed in every sample.

The aim of this research was to study the changes in the emission of these Sr_2_MgSi_2_O_7_:Eu^2+^, Dy^3+^ glass-ceramics obtained by sintering and crystallisation of glass powders as a function of the dopant concentrations. Glasses of the same base composition (55SiO_2_-27SrO-18MgO, mol%.) with increasing Eu_2_O_3_ and Dy_2_O_3_ concentration were melted in a gas furnace, and their corresponding glass-ceramics were obtained after suitable thermal treatment. A complete study of the thermal, structural, and optical properties allows the selection of an optimum dopant level content in order to achieve higher persistence times.

## 2. Experimental Procedure

### 2.1. Glasses and Glass-Ceramics Preparation

Glasses of the same base composition 55SiO_2_-27SrO-18MgO mol%. doped with different amounts of Eu_2_O_3_ and Eu_2_O_3_/Dy_2_O_3_ were prepared by melt-quenching. The employed raw materials were SiO_2_ sand (Saint-Gobain, 99.6%), SrCO_3_ (Alfa Aesar, 97.5%), MgO (PanReac, 98%), Eu_2_O_3_ (Alfa Aesar, >99.9%), and Dy_2_O_3_ (Alfa Aesar, >99.9%). Batches of 100 g were mixed and stirred in a Turbula mixer for one hour to achieve homogenisation. The glasses were melted in a gas furnace in air using an alumina–zirconia–silica (AZS; Al_2_O_3_-ZrO_2_-SiO_2_) crucible. The temperature was maintained at 1300 °C for 15 min, followed by an increase to 1550 °C for 1 h, with final heating to 1600 °C. As soon as the furnace reached 1600 °C (without dwell time), the glass was poured into water to obtain a glass frit.

The frits were milled in acetone, and the powders were sieved below 20 µm. Cylindrical pellets (Ø = 2.5 cm) were prepared by pressing the powder into a die (1600 Kp for 3 min) before firing in an electric furnace at 1100 °C (heating and cooling rate 10°C/min) for 1 min in the air atmosphere.

Table 1 summarises the studied glass-ceramic samples, whose names indicate that they are glass-ceramic (GC) samples and the dopant content in mol%. The glasses and glass-ceramics were first observed with the naked eye under the light of a UV lamp at 365 nm, which served as an excitation source. All original glass frits showed red emission under the excitation source, and most glass-ceramics showed blue emission under the excitation source. As widely discussed in a previous paper [26], the blue luminescence is associated with the T_2g_→^8^S_7/2_ transition of Eu^2+^ ions in the Sr_2_MgSi_2_O_7_ crystals. The table also lists the persistence that the materials showed at room temperature and at 0 °C once the excitation source was switched off. After the blue emission ceased, there was still white emission remaining for longer times, in the case of the higher blue persistence samples—GC-1Eu-0.5Dy and GC-1Eu-1Dy—the white emission reached 152 s and 261 s, respectively [27].

### 2.2. Thermal and Structural Characterisation

DTA curves were recorded with a SETARAM Setsys Evolution instrument, using glass powder of particle size < 20 µm with heating rates 2, 5, 10, 20, and 30 °C/min up to 1200 °C.

An EM 201 side-view hot-stage microscope (HSM) with image analysis and 1750/15 Leica electrical furnace was used to determine the sintering and flow behaviour of the glass powders. Details of the equipment have been reported previously [28]. Measurements were conducted in air at a heating rate of 10 °C/min on powder samples with particle sizes < 20 µm. The temperature was measured with a Pt/Rh (6/30) thermocouple placed under the alumina support and in contact with it. The changes in the area of the samples are associated with different processes and correspond to points of viscosity that were previously determined [29].

The density of glass-ceramics was measured according to the Archimedes method using distilled water.

The glass-ceramic pellets were milled and sieved to a particle size lower than 60 µm and initially characterised by X-ray diffraction (Bruker D8 Advance, Massachusetts, USA) in the range of 10–70° 2θ, with a step size of 0.02°, employing CuKα_1_ radiation (λ = 1.54056 Å).

Scanning electron microscopy (SEM) of the glass-ceramic samples was performed on a Hitachi S-3000N microscope, Tokyo, Japan equipped with a vacuum chamber. The instrument is equipped with secondary electron (SE) and backscattering electron (BSE) detectors, as well as an Oxford Instruments energy-dispersive X-ray spectroscopy (EDX) analyser, model INCAx-sight, and allows samples to be inclined at 90°.

Scanning electron microscopy–cathodoluminescence (SEM–CL) of the same selected glass-ceramic samples was performed on a Hitachi S-3000N microscope equipped with a vacuum chamber, on excitation with an electron beam of voltage 15–25 kV and a filament intensity of 100 µÅ. The emission spectra were recorded employing a fiber spectrometer with a charge-coupled device (CCD) through an optical fiber, and the corresponding luminescence photographs, with an ordinary camera. The instrument is equipped with secondary electron (SE) and backscattered electron (BSE) detectors, an energy-dispersive X-ray spectroscope (EDX) Quantax (model XFlash 6I30, Bruker, Massachusetts, USA), and a cathode-luminescence system (CHROMA-CL2 Gatan, Pleaston, CA, USA). Samples were compositionally accurate within the uncertainty in the EDX (~1%). Additionally, spectrally resolved CL measurements were carried out at 80 K on a MONO-CL2 system (Gatan, Pleaston, CA, USA) attached to a field-emission scanning electron microscope (FE-SEM, ZeissLEO 1530, Jena, Germany). Detection was performed with a photomultiplier for panchromatic images, and a Peltier cooled Si-CCD for spectrally resolved images.

### 2.3. Optical Characterisation

The glass-ceramic pellets were firstly observed under UV light (18 W (λ = 365 nm), I = 0.025 mA/cm^2^) to check for emission and persistence of phosphorescence.

Room temperature emission and excitation spectra, as well as temporal decays, were measured in an Edinburgh FS5 Spectrofluorometer equipped with a 150 W Xenon lamp. The emission was detected by the Hamamatsu R928P photomultiplier. The persistent luminescence decays were obtained after illumination of the samples in the UV (354 nm) with the Xe lamp for 10 min. After that, the illumination was blocked and the luminescence decays were monitored for 600 s with a resolution of 0.5 s.

## 3. Results and Discussion

### 3.1. Thermal and Structural Properties

**Figure 1:**
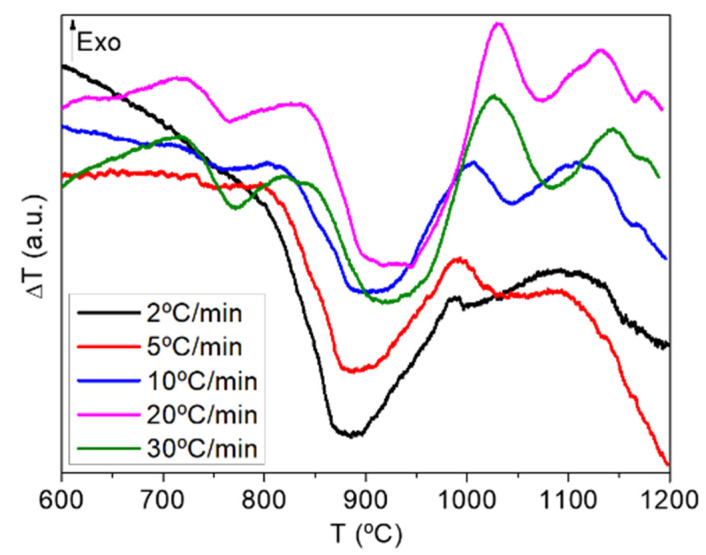
DTA curves of the undoped glass with different heating rates (ø < 20 µm).

The effect of different heating rates on the crystallisation temperatures obtained from DTA curves is illustrated in Figure 1 and Table 2 for the G-undoped glass. On increasing the heating rate, the nucleation time decreased and, as a result, the crystallisation peaks appeared at a higher temperature.

**Figure 2:**
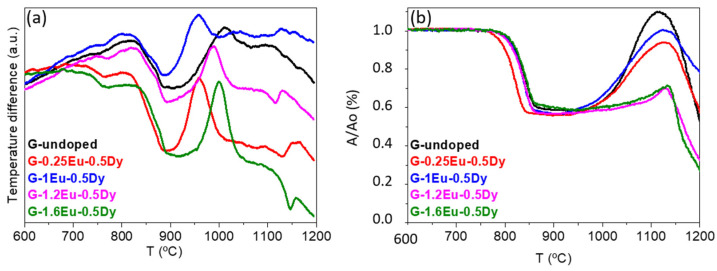
(**a**) DTA and (**b**) HSM curves of G-undoped, G-0.25Eu-0.5Dy, G-1Eu-0.5Dy, G-1.2Eu-0.5Dy, and G-1.6Eu-0.5Dy glasses with different dopant concentrations. Heating rate: 10 °C/min.

Figure 2a and Table 3 represent the DTA results for compositions doped with different Eu/Dy concentrations and a particle size < 20 µm. The addition of dopant resulted in a higher glass transition temperature (T_g_). Variations in the crystallisation of the samples were observed when changing the number of dopants. In the co-doped samples (with 0.5%Dy), crystallisation was observed to slow down with increasing europium concentration. In the co-doped samples (with 1%Dy), the same occurred—i.e., crystallisation was slower as the number of europium increased. Figure 2b shows the HSM results for glass powders with different dopant concentrations; sintering and flow temperatures are given in Table 4. The softening temperature (T_S_) was between 780 and 840 °C for all samples. In general, the samples became spherical around 1000–1100 °C, but sample G-1Eu-0.5Dy did not become spherical until 1140 °C. All samples reached half-ball temperature (T_HB_) above 1150 °C and then flowed.

The combination of DTA and HSM results indicated that heat treatment of the glass powders up to 1100 °C can provide suitable sintering, crystallisation, and flow stages, thus allowing the production of glass-ceramic in bulk or enamel form as possible final products.

The densities of the glass-ceramics were measured by the Archimedes method, but for the density of the glass samples, we adopted the value of the melts in an electric furnace as a reference, since, as previously mentioned for the gas furnace, the glass melted in a glass frit form. The average density of the glass melted in the electric furnace was about 3.31 g·cm^−3^, and the density of glass-ceramics is presented in Table 5. The theoretical density of the main crystalline phase, Sr_2_MgSi_2_O_7_, was 3.7 g·cm^−3^, while the highest density obtained for the glass-ceramics was 3.27 g·cm^−3^ for GC-1Eu-0.5Dy. Although it was not quantified, there was a high residual porosity in some of the samples.

### 3.2. X-ray Diffraction

**Figure 3:**
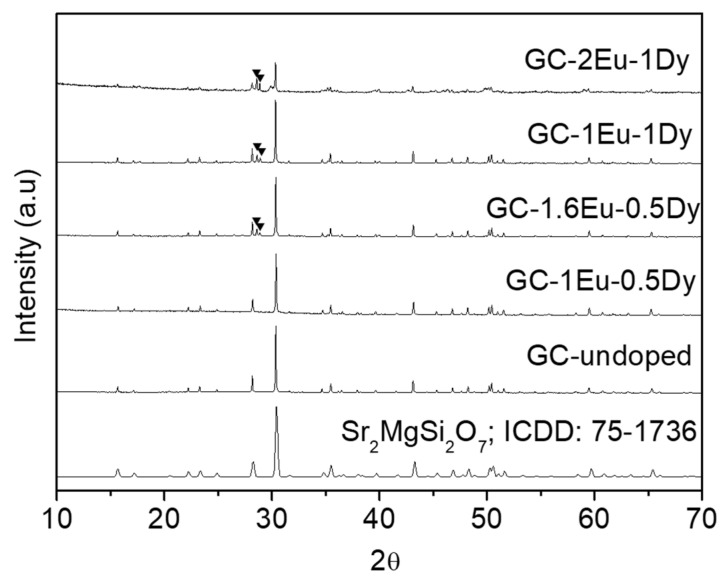
XRD patterns of glass-ceramic samples treated at 1100 °C for 1 min.

Figure 3 shows the X-ray diffraction patterns of some of the glass-ceramics treated at 1100 °C for 1 min. As can be seen in sample GC-undoped and GC-1Eu-0.5Dy, only the Sr_2_MgSi_2_O_7_ phase (ICDD: 75–1736) appeared. The increase in the number of dopants, both europium and dysprosium, resulted in the appearance of two small peaks, not corresponding to the akermanite phase. These unidentified peaks are shown in samples GC-1.6Eu-0.5Dy, GC-1Eu-1Dy, and GC-2Eu-1Dy in Figure 3.

### 3.3. SEM and CL–SEM

**Figure 4:**
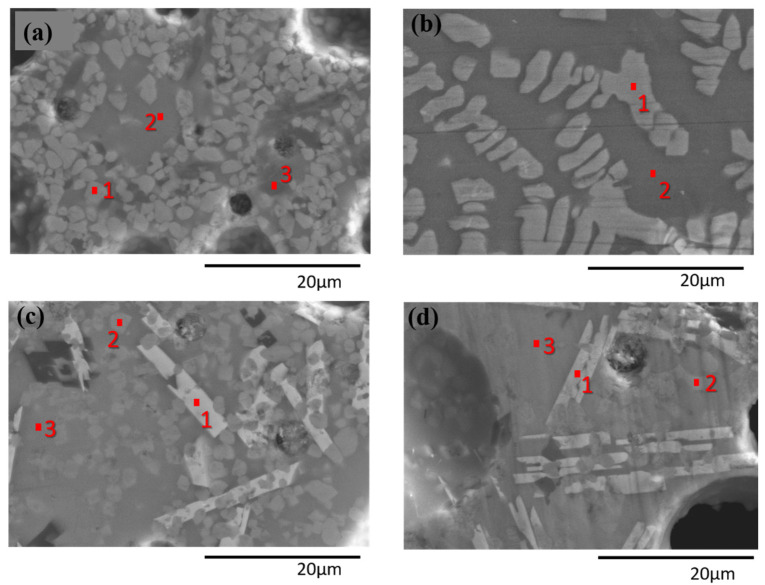
SEM images of (**a**) GC-undoped, (**b**) GC-1Eu-0.5Dy, (**c**) GC-1Eu-1Dy, and (**d**) GC-2Eu-1Dy glass-ceramics.

An SEM analysis of the polished surface of glass-ceramic samples was carried out. No increase in the size of the crystals was observed, which in most glass-ceramic samples, were rounded crystals (2 µm) in the Sr_2_MgSi_2_O_7_ phase. In GC-1Eu-0.5Dy, the crystals appeared with a different shape: they grew in a dendritic form, and their size was larger (4–6 µm). Figure 4 shows SEM images of GC-undoped, GC-1Eu-0.5Dy, GC-1Eu-1Dy, and GC-2Eu-1Dy. It is possible to appreciate the very different microstructures. In the GC-undoped (Figure 4a), only one type of crystal (point 1) corresponding to the akermanite phase was observed, and the crystals were small and rounded. Points 2 and 3 correspond to the residual glassy phase. The sample GC-1Eu-0.5Dy (Figure 4b) showed dendritic form crystals (more than 10 µm) that also corresponded to the akermanite phase (point 1). In comparison, in GC-1Eu-1Dy (Figure 4c) and GC-2Eu-1Dy (Figure 4d), there were two types of crystals; the crystals corresponding to the akermanite phase were again small (2 µm) and rounded (point 2 in both cases). In addition, these glass-ceramics presented elongated white crystals (point 1 in both cases), corresponding to the unidentified phase, the chemical analysis of which is shown in Table 6; this phase was rich in SiO_2_ and SrO. Point 3 corresponds to the residual glassy phase. In the sample GC-2Eu-1Dy, these white crystals predominated, although some small crystals of the Sr_2_MgSi_2_O_7_ phase could also be observed. These results coincide with those obtained previously by X-ray diffraction.

EDX analysis of the original glass frits was carried out to determine if there was alumina or zirconia incorporation from the AZS crucible (Table 7). All glass compositions contained some alumina percentage. The EDX analysis of the previously mentioned points in Figure 4 in the glass-ceramic samples is shown in Table 7.

**Figure 5:**
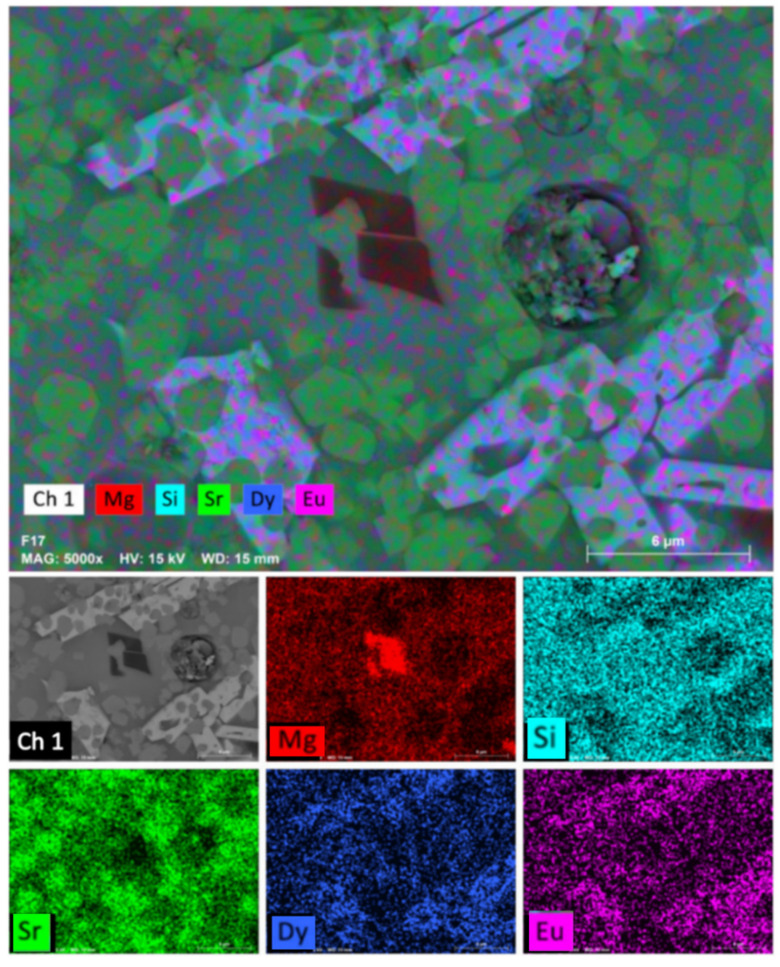
Elemental mapping of GC-1Eu-1Dy glass-ceramic.

Figure 5 shows an elemental map of GC-1Eu-1Dy in which it can be seen again how europium and dysprosium are concentrated in the white crystals, coinciding with the EDX analysis of the same sample. In the gray crystals, the elements corresponding to the akermanite phase, strontium, silicon, and magnesium were clearly found.

**Figure 6:**
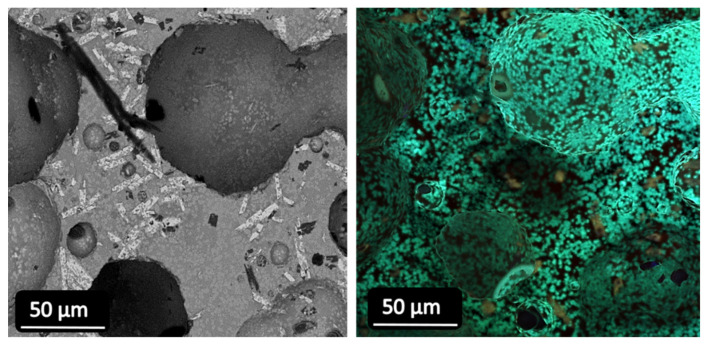
SEM-CL images of the polished surface of GC-1Eu-1Dy glass-ceramic. SEM images (×2.0 k), CL images (×1.0 k).

The glass-ceramic microstructure and the crystals responsible for the emission can be observed by means of the panchromatic CL images in Figure 6. The images correspond to the surface of a polished pellet sample of GC-1Eu-1Dy, with the microscope focused on the central part of the pellet. In the images presented below, SEM (black and white) and panchromatic (green) images corresponding to the same area of the pellet are compared. The green emission originated from all grey microcrystals, while the residual glassy phase remained dark.

### 3.4. Optical Properties

**Figure 7:**
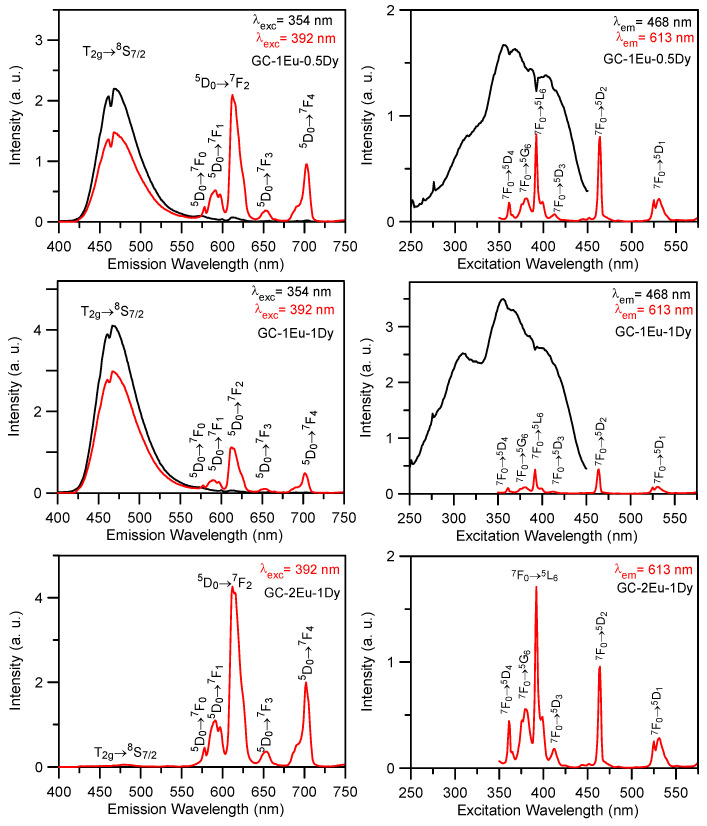
(**Left**) Room temperature emission spectra obtained under excitation at 354 nm (Eu^2+^) (black line) and 392 nm (Eu^3+^) (red line) for the GC-1Eu-0.5Dy, GC-1Eu-1Dy and GC-2Eu-1Dy glass-ceramics; (**right**) excitation spectra obtained by collecting the luminescence at 468 nm (black line) and 613 nm (red line).

The emission and excitation spectra of the GCs demonstrated the presence of Eu^2+^ and Eu^3+^ in the single and co-doped samples. Figure 7 shows the emission and excitation spectra for some of the glass-ceramic samples obtained in this research. On the left are the emission spectra measured in the 400–750 nm range, under excitation at 354 nm (Eu^2+^) and 392 nm (Eu^3+^). As can be seen for the GC-1Eu-0.5Dy and GC-1Eu-1Dy glass-ceramic samples, under 354 nm excitation, the spectra showed a broad band centred at 468 nm, and attributed to the T_2g_→^8^S_7/2_ transition of Eu^2+^, together with a weak red emission at 613 nm, corresponding to the Eu^3+ 5^D_0_→^7^F_2_ transition. However, under 392 nm excitation, in addition to the blue emission, the spectra clearly showed Eu^3+^ emissions, corresponding to the ^5^D_0_→^7^F_0,1,2,3,4_ transitions. In the case of the co-doped GC sample with 2Eu-1Dy, the main contribution to the emission spectrum corresponded to the f–f transitions of Eu^3+^ ions. The peak positions remained unchanged for all samples. The highest intensity ratio between the blue emission of Eu^2+^ and the red emission at 613 nm of Eu^3+^ under 392 nm corresponded to 1Eu-1Dy, in agreement with the highest persistent luminescence from this sample.

In the case of Eu^3+^ ions, the intensity of the ^5^D_0_→^7^F_2_ hypersensitive transition was strongly affected by the host matrix, and the influence of the local field on the emission spectra of Eu^3+^ can be described by the relative intensities of the ^5^D_0_→^7^F_2_ and ^5^D_0_→^7^F_1_ emissions. In these GC samples, in all cases, the intensity of the electric-dipole ^5^D_0_→^7^F_2_ transition was much higher than that of the ^5^D_0_→^7^F_1_ magnetic–dipole transition, which indicated an asymmetric environment for Eu^3+^ ions [30].

On the right are the excitation spectra monitored at 468 nm and 613 nm for the three glass-ceramic samples. For all samples, except for the GC-2Eu-1Dy co-doped sample, the excitation spectra obtained by collecting the luminescence at 468 nm (Eu^2+^) showed broadband, with its maximum at 354 nm, whereas the spectra obtained by monitoring the luminescence at 613 nm (Eu^3+^) displayed the f–f intra-configurational transitions corresponding to the ^7^F_0_→^5^D_4,3,2_ (361, 412, and 464 nm, respectively), ^7^F_0_→^5^G_6_ (381 nm), ^7^F_0_→^5^L_6_ (392 nm), and ^7^F_0_→^5^D_1_ (531 nm) transitions.

Focusing on the intensities, it was observed that, on the one hand, the GC-1Eu-1Dy sample had a high emission intensity, which made it a very efficient sample, followed by the GC-1.2Eu-0.5Dy (not shown in Figure 7) and GC-1Eu-0.5Dy glass-ceramics. On the other hand, for the GC-2Eu-1Dy sample, only Eu^3+^ emission was observed; this agrees with the results thus far because no blue emission was observed in the sample GC-2Eu-1Dy.

**Figure 8:**
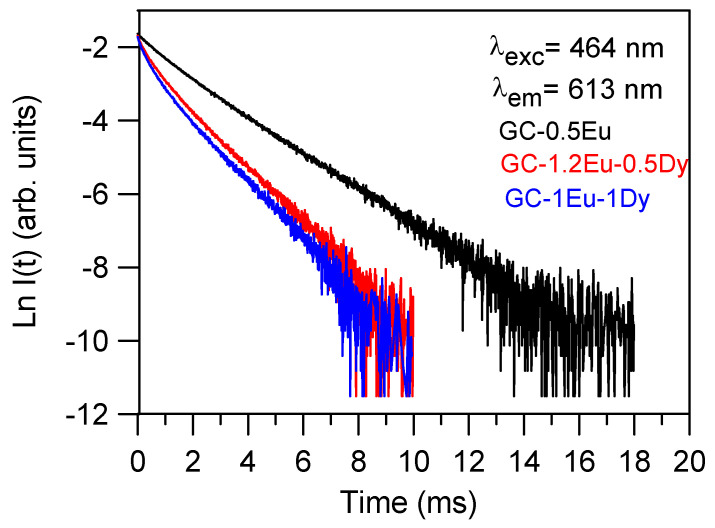
Experimental decays from level ^5^D_0_ (Eu^3+^) obtained by exciting at 464 nm collecting the luminescence at the ^5^D_0_→^7^F_2_ emission (613 nm).

The decays of the ^5^D_0_ (Eu^3+^) level were obtained for all samples by exciting at 464 nm and collecting the luminescence at the maximum of the ^5^D_0_→^7^F_2_ emission (613 nm). Figure 8 shows, as an example, the decays for three of the glass-ceramic samples studied. As can be seen, the decays deviated from a single exponential function and decreased with the increasing dopants concentration. Table 8 shows the lifetimes of all the glass-ceramic samples. The lifetime values correspond to the average lifetime defined by $\tau =\frac{\int_{0}^{\infty }tI\left(t\right)dt}{\int_{0}^{\infty }I\left(t\right)dt}$, where *I (t)* represents the luminescence intensity at time *t* corrected for the background.

According to these results, the longest decay would be for samples GC-0.5Eu and GC-2Eu-1Dy, which agreed with the rest of the characterisations; sample GC-0.5Eu did not persistently show blue emission, and sample GC-2Eu-1Dy only showed red emission (as seen in the SEM of Figure 4, this sample was predominated by crystals that were not of the akermanite phase). The sample with the fastest Eu^3+^ decay was GC-1Eu-1Dy, which seems reasonable since it was the sample with the longest persistence time of blue emission. It was followed by GC-1Eu-0.5Dy, GC-1.2Eu-0.5Dy, and GC-1.6Eu-0.5Dy.

**Figure 9:**
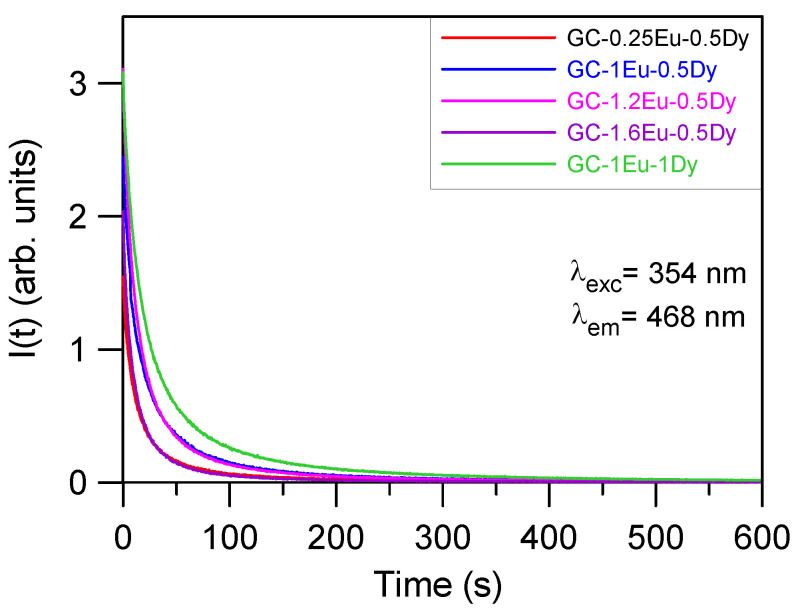
Persistent luminescent decays at 468 nm from Eu^2+^, excited for 10 min at 354 nm for the co-doped samples.

To obtain the persistent luminescence decays of the blue emission at 468 nm, the samples were excited for 10 min at 354 nm. After 10 min excitation, the illumination was blocked, and the kinetics of the afterglow was further monitored for 600 s. Figure 9 shows the decays for co-doped samples exhibiting blue luminescence. The decays can be described by two single exponential functions. The obtained lifetimes corresponding to the two components (τ_1_ and τ_2_) of the decays are presented in Table 9. As can be seen, co-doping with Dy^3+^ increased the persistence of luminescence, the best result of which was observed in the one corresponding to the GC-1Eu-1Dy sample.

As seen in Table 1 at RT, the sample with the longest persistence of blue emission with the naked eye was GC-1Eu-1Dy, which agrees with the data in Table 9, according to which the longest lifetime was for the GC-1Eu-1Dy sample. The next longest persistence samples were the glass-ceramic samples GC-1Eu-0.5Dy and GC-1.2Eu-0.5Dy.

## 4. Conclusions

Sr_2_MgSi_2_O_7_ phosphor-based glass-ceramics doped with increasing Eu_2_O_3_ and Dy_2_O_3_ concentrations were prepared from sintering and crystallisation of glass powders.

The prepared Eu-doped and Eu/Dy co-doped glasses showed red emissions; furthermore, after heat treatment, the corresponding glass-ceramics emitted blue under UV light excitation, except for the composition 2Eu-1Dy (mol%.) which kept the red emission. The composition 1Eu-1Dy (mol%.) showed the highest persistence results for blue emission. The different dopant concentrations influenced the final chemical composition, which considerably affected the crystallisation process, resulting in Sr_2_MgSi_2_O_7_ glass-ceramics with very different microstructures, which, in turn, had a significant influence on the luminescence and persistence properties.

The emission spectra of the glass-ceramics showed characteristics attributable to Eu^2+^ and Eu^3+^ cations. The glass-ceramics co-doped with 1Eu-1Dy (mol%.) provided spectra with a higher signal intensity, as well as a longer Eu^2+^ persistent blue luminescence. The glass-ceramic co-doped with 2Eu-1Dy (mol%.) only showed the emission spectrum corresponding to Eu^3+^, in agreement with the red emission seen under UV light and the structural characterisation.

## Figures and Tables

**Table 1:** Glass-ceramics nomenclature and persistent blue emission information. Measurements were repeated 3–4 times with an error of about 3 s.

Sample Name	Emission under UV Lamp (365 nm)	Persistent Blue Emission at RT (Time)	Persistent Blue Emission at 0 °C (Time)
GC-0.5Eu	Blue	-	-
GC-0.25Eu-0.5Dy	Blue	22.96 s	102.52 s
GC-1Eu-0.5Dy	Blue	75.90 s	268.29 s
GC-1.2Eu-0.5Dy	Blue	39.07 s	146.57 s
GC-1.6Eu-0.5Dy	Blue	8.39 s	15.57 s
GC-1Eu-1Dy	Blue	83.33 s	272.17 s
GC-2Eu-1Dy	Red	-	-

**Table 2:** T_g_, T_x_, and T_c_ of the undoped glass (ø < 20 µm) determined by DTA.

Heating Rate	T_g_(°C) ± 7	T_x_(°C) ± 9	T_c1_(°C) ± 9	T_c2_(°C) ± 9
2 °C/min	-	878	986	1082
5 °C/min	720	891	991	1085
10 °C/min	720	901	1006	1106
20 °C/min	722	909	1029	1129
30 °C/min	717	928	1027	1141

**Table 3:** T_g_, T_x_, and T_c_ of all glass (ø < 20 µm) samples determined with DTA.

Sample	T_g_(°C) ± 7	T_x_(°C) ± 9	T_c_(°C) ± 9
G-undoped	720	901	1006
G-0.5Eu	721	887	967
G-0.25Eu-0.5Dy	727	895	959
G-1Eu-0.5Dy	728	897	960
G-1.2Eu-0.5Dy	746	911	989
G-1.6Eu-0.5Dy	737	922	1000
G-1Eu-1Dy	743	897	961
G-2Eu-1Dy	748	917	1000

**Table 4:** Sintering and flow temperatures of glass (ø <20 µm) samples determined with HSM.

Sample	T_FS_(°C) ± 10	T_MS_(°C) ± 10	T_S_(°C) ± 10	Sphere(°C) ± 10	T_HB_(°C) ± 3	T_F_(°C) ± 3
G-undoped	790	910	940	1000	1159	1190
G-0.5Eu	780	890	920	1020	1190	1197
G-0.25Eu-0.5Dy	780	1010	1030	1090	1170	1190
G-1Eu-0.5Dy	820	880	1020	1100	1174	1200
G-1.2Eu-0.5Dy	810	890	990	1100	1160	1176
G-1.6Eu-0.5Dy	840	920	1030	1090	1148	1159
G-1Eu-1Dy	830	950	1100	1140	1160	1180
G-2Eu-1Dy	840	910	948	1000	1184	1195

**Table 5:** Glass-ceramic densities.

Sample	Glass-Ceramic Density ± 0.01 (g/cm^3^)
GC-undoped	3.11
GC-0.5Eu	3.17
GC-0.25Eu-0.5Dy	3.19
GC-1Eu-0.5Dy	3.27
GC-1.2Eu-0.5Dy	3.26
GC-1.6Eu-0.5Dy	3.19
GC-1Eu-1Dy	3.22
GC-2Eu-1Dy	3.21

**Table 6:** Compositional analysis (mol%.) of GC-Undoped, GC-1Eu-0.5Dy, GC-1Eu-1Dy, and GC-2Eu-1Dy glass-ceramics as determined by EDX corresponding to the points shown in Figure 4.

	SiO_2_	SrO	MgO	Al_2_O_3_	Eu_2_O_3_	Dy_2_O_3_
GC-Undoped						
Theoretical glass composition	55.0	27.0	18.0	-	-	-
1 Sr_2_MgSi_2_O_7_	40.9	39.5	17.3	2.4	-	-
2	62.1	21.1	11.7	5.1	-	-
3	60.1	21.4	16.8	1.0	-	-
GC-1Eu-0.5Dy						
Theoretical glass composition	54.1	26.6	17.7	0	0.9	0.5
1 Sr_2_MgSi_2_O_7_	40.9	38.1	17.9	2.0	0.5	0.2
2	56.6	20.4	16.2	4.0	1.7	0.8
GC-1Eu-1Dy						
Theoretical glass composition	53.9	26.4	17.6	0	0.9	0.9
1	50.8	34.9	6.8	1.9	2.7	2.7
2 Sr_2_MgSi_2_O_7_	41.1	39.5	18.0	0.8	0.3	0.0
3	62.1	23.2	10.4	2.3	0.8	1.0
GC-2Eu-1Dy						
Theoretical glass composition	53.4	26.2	17.4	0	1.9	0.9
1	64.0	26.1	1.7	2.1	4.3	1.6
2 Sr_2_MgSi_2_O_7_	39.7	38.4	20.0	1.3	0.4	0.0
3	63.8	15.7	14.2	3.4	1.7	0.9

**Table 7:** EDX analysis of glass frits (mol%.). Samples were compositionally accurate within the uncertainty in the EDX (~1%).

Sample Name	SiO_2_	SrO	MgO	Al_2_O_3_
G-undoped	51.7	29.2	16.8	2.4
G-0.5Eu	56.7	24.4	17.3	1.6
G-0.25Eu-0.5Dy	50.8	27.3	18.2	3.8
G-1Eu-0.5Dy	56.7	24.8	17.6	0.8
G-1.2Eu-0.5Dy	56.4	23.6	18.4	1.5
G-1.6Eu-0.5Dy	54.2	22.6	20.7	2.5
G-1Eu-1Dy	56.5	22.6	17.6	3.3
G-2Eu-1Dy	55.9	23.3	18.9	1.9

**Table 8:** Experimental lifetimes from level ^5^D_0_ (Eu^3+^) obtained by exciting at 464 nm collecting the luminescence at the ^5^D_0_→^7^F_2_ emission (613 nm).

GC-05Eu	GC-0.2Eu-0.5Dy	GC-1Eu-0.5Dy	GC-1.2Eu-0.5Dy	GC-1.6Eu-0.5Dy	GC-1Eu-1Dy	GC-2Eu-1Dy
1.28 ms	1.27 ms	0.91 ms	0.93 ms	0.97 ms	0. 89 ms	1.06 ms

**Table 9:** Lifetimes of the persistent luminescence kinetics excited at 354 nm for the co-doped samples.

GC-0.25Eu-0.5Dy	GC-1Eu-0.5Dy	GC-1.2Eu-0.5Dy	GC-1.6Eu-0.5Dy	GC-1Eu-Dy1
τ_1_ = 9.4 s τ_2_ = 52.8 s	τ_1_ = 10.3 s τ_2_ = 57.9 s	τ_1_ = 10.3 s τ_2_ = 53.6 s	τ_1_ = 7.7 s τ_2_ = 39.7 s	τ_1_ = 14.8 s τ_2_ = 77.5 s

## Data Availability

Not applicable.
